# Ultraminiaturized Microfluidic Electrochemical Surface‐Enhanced Raman Scattering Chip for Analysis of Neurotransmitters Fabricated by Ship‐in‐a‐Bottle Integration

**DOI:** 10.1002/smsc.202200093

**Published:** 2023-01-29

**Authors:** Shi Bai, Ying Ma, Kotaro Obata, Koji Sugioka

**Affiliations:** ^1^ Advanced Laser Processing Research Team RIKEN Center for Advanced Photonics 2-1 Hirosawa, Wako Saitama 351-0198 Japan; ^2^ School of Material Science and Engineering Hebei University of Science and Technology Shijiazhuang 050018 China; ^3^ Academy of Artificial Intelligence Beijing Institute of Petrochemical Technology No.19 North Qingyuan Road, Daxing District Beijing 102617 China

**Keywords:** hybrid laser processing, microfluidic chips, neurotransmitters, ship-in-a-bottle integration

## Abstract

Electrochemical surface‐enhanced Raman scattering (EC‐SERS) is a promising technique for the diagnosis of trace amounts of neurotransmitters, because it can elucidate neurotransmitters’ behavior on electrodes to deduce their functions in the human body. However, the current EC‐SERS devices need several tens of milliliters of analyte solution to collect enough signal for analysis. Miniaturization of EC‐SERS devices is crucial for the early diagnosis of disease and point‐of‐care testing. Herein, a new type of EC‐SERS sensor based on 3D microfluidic chips for the analysis of neurotransmitters in ultrasmall volumes is proposed. The microfluidic EC‐SERS chip is fabricated by a ship‐in‐a‐bottle technique based on hybrid laser processing. The working electrode is modified using silver/zinc oxide materials, enabling the formation of a unique “candy apple” structure. To assess the fabricated microfluidic EC‐SERS chips, ascorbic acid is analyzed using the ingenious microfluidic EC‐SERS chips to elucidate its redox reaction by EC‐SERS spectroscopy. Significantly, a sub‐10 μL volume of analyte solution is sufficient for EC‐SERS analysis, which is several orders smaller in volume than the requirements of current EC‐SERS devices. The unprecedented microfluidic EC‐SERS chips fabricated by the ship‐in‐a‐bottle integration technique can be used in portable and smart analyzers for next‐generation biomedicines and catalysts.

## Graphical abstract

A microfluidic electrochemical surface‐enhanced Raman scattering (EC‐SERS) chip is fabricated by a ship‐in‐a‐bottle technique based on hybrid laser processing. The working electrode is modified using silver/zinc oxide materials, enabling the formation of a unique “candy apple” structure. A sub‐10 μL volume of analyte solution is sufficient for EC‐SERS analysis.

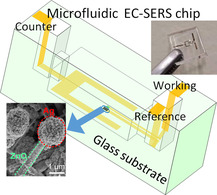

## 1 Introduction

Neurotransmitters are signaling molecules and often called the chemical messengers of the body, and are vital for neural activities and the metabolism of cells. Trace analysis of neurotransmitters in body fluids is critical for the recognition and diagnosis of diseases of the human nervous system in the early stages, because changes in neurotransmitter concentrations in the nervous system may cause serious diseases. It has been found that if neurotransmitters are dysfunctional, glutamate and *γ*‐aminobutyric acid can give rise to Alzheimer's disease;^[^ ^1^ ^]^ and dopamine may cause Parkinson's disease.^[^ ^2^ ^]^ However, these nerve diseases at early stages are difficult to recognize by medical imaging methods such as computerized tomography and magnetic resonance imaging, because the variations of neurotransmitters are at the sub‐micromolar level. Alternatively, electrochemical surface‐enhanced Raman scattering (EC‐SERS) is anticipated to be an effective analysis method for trace amounts of neurotransmitters, since it provides the advantages of non‐contact measurement and rapid detection with high sensitivity.^[^ ^3^ ^]^

EC‐SERS can take advantage of the capabilities of both EC and SERS measurements. Thanks to the ultrahigh sensitivity attributed to SERS, the analysis can be conducted at trace levels (usually the nanomolar level) to elucidate the details of chemical reactions. Additionally, the trace amounts of intermediates produced in chemical reactions can be investigated and analyzed due to the EC functionality. Therefore, unlike regular EC and SERS, the advantage of the EC‐SERS technique is that neurotransmitters’ behavior, such as the investigation of metabolism, changes in the molecular structure during redox reactions, and the identification of intermediates, can be elucidated by EC‐SERS spectroscopy, which is useful for deducing the functions of neurotransmitters in the human body.^[^ ^4^ ^]^

Among the methods for fabricating EC chips including EC‐SERS chips, a screen‐printing technique is most commonly used because it offers an economical, reproducible, and robust platform. The EC chip is typically composed of a three‐electrode system on an inert substrate, which involves a working electrode, a reference electrode, and a counter electrode.^[^ ^5^ ^]^ Briefly, the three electrodes are fabricated by deposition of multilayer metal thin films on the substrate using a mesh mask fabricated by screen‐printing. The ink/paste is ejected to deposit on the open regions of the mesh for the formation of the electrodes on the substrate. The ink/paste contains metallic pastes (gold, silver, or platinum) or carbon materials (carbon black, nanotubes, or graphene) in solvents. By repeating the screen‐printing process, the three electrodes can be fabricated layer by layer according to the designed structure. The printed electrodes are then cured by an annealing process to volatilize the solvents. Screen‐printed electrodes (SPEs) are widely used for EC analysis. EC‐SERS chips are, however, not well developed and rarely commercially available, because the SERS substrates need to be fabricated on the electrode by an additional process. Generally, EC chips with SPEs have a relatively large size (a few cm^2^) and it is difficult to miniaturize the chip size, due to the limited fabrication resolution. This low resolution is ascribed to the spreading of ink during the printing process, which is hard to overcome. In addition, screen printing cannot fabricate complicated 3D and hybrid structures, which is essential for the improvement of SERS sensitivity. For cost‐effectiveness, high efficiency, low environmental load, and high speed for analysis, reducing the volume of the analyte solution used is important, so that miniaturization of the chip is required. More importantly, the reduction of sample volume benefits real clinical applications and point‐of‐care testing. For example, cerebrospinal fluid (CSF) analysis is a diagnostic tool for central nervous system diseases, such as Parkinson's disease, Alzheimer's disease, depressive disorders, and others.^[^ ^6^ ^]^ In clinical practice, it takes ≈30 min to collect ≈5 mL of CSF sample, during which the patient suffers from pain. Therefore, minimizing the CSF volume necessary for analysis not only relieves the suffering of patients, but also reduces the effort needed by medical staff to collect CSF. To this end, in this article, we propose a new strategy using a ship‐in‐a‐bottle technique based on hybrid laser processing, which can create ultraminiaturized microfluidic EC‐SERS chips with a size of a few mm^2^. The ship‐in‐a‐bottle technique has an advantage over the high‐temperature bonding technique. Specifically, the high‐temperature bonding is somewhat burdensome in terms of alignment as well as an increase in process steps. Additionally, the high temperature for bonding may deteriorate the characteristics of EC‐SERS chips. Thus, the ship‐in‐a‐bottle technique is promising to create high‐performance EC‐SERS chips with reduced process steps. Consequently, the volume of analyte solution necessary for analysis is tremendously reduced compared with the current EC‐SERS chips with SPEs.

Hybrid laser processing, in which different types of laser processing are successively implemented, offers higher flexibility and versatility than conventional laser processing, because it can compensate for the drawbacks of each processing step while maintaining the original performance to create much more complicated structures with enhanced functionalities. More importantly, hybrid laser processing allows us to directly integrate functional micro‐components inside closed microfluidic channels (ship‐in‐a‐bottle integration). For example, Wu et al. reported that cancer cells were able to be identified in a flow of cells, and the number of cells was counted using a biochip in which polymer micro/nanocomponents were integrated into 3D microchannels by femtosecond laser‐assisted etching (FLAE) and subsequent femtosecond (fs) laser two‐photon polymerization.^[^ ^7^ ^]^ Xu et al. demonstrated the manipulation of microorganisms in a 3D glass microchannel integrated with metal electrodes by FLAE followed by fs laser‐assisted selective metallization.^[^ ^8^ ^]^ Ship‐in‐a‐bottle integration using a hybrid fs laser was also used to fabricate 3D microfluidic SERS chips for the trace detection of toxic substances in real‐time^[^ ^9^ ^]^ and attomolar sensing of biomaterials.^[^ ^10^ ^]^

Compared to the screen‐printing technique, hybrid laser processing greatly improves the fabrication resolution, with features down to ≈100 nm.^[^ ^11^ ^]^ The noncontact scheme of ship‐in‐a‐bottle integration enables the formation of the electrode system in a microscale glass chamber, which allows us to conduct both EC and SERS analysis with sub‐10 μL analyte solution volume in the microfluidic chamber. In contrast, an SPE device based on a planar chip requires a vessel/cell to immerse the electrodes that can accommodate ≈100 mL solution. The large volume of solution necessary for analysis causes difficulties in some real applications, such as the abovementioned CSF analysis and tear analysis, where it is difficult to collect sufficient volume in a single sampling. Meanwhile, in addition to minimizing the sample volume, ship‐in‐a‐bottle integration enables us to fabricate customized microfluidic EC‐SERS devices with additional functions on demand.

In this article, we further advance the ship‐in‐a‐bottle integration technique by integrating new types of laser processing to realize a microfluidic EC‐SERS chip. Specifically, after FLAE and fs laser‐assisted selective metallization, the reference electrode is formed by a treatment using fs‐laser‐induced localized melting of silver nanoparticles on gold substrates, while the silver/zinc oxide modified working electrode is generated by continuous wave (CW)‐laser‐induced hydrothermal growth of zinc oxide nanowires followed by CW laser reduction to produce silver nanoparticles for SERS sensing. In this work, the microfluidic EC‐SERS chip fabricated by the advanced ship‐in‐a‐bottle integration technique is applied for the analysis of neurotransmitters as a demonstration to show the analytical capability of the technique utilizing extraordinarily smaller volumes of analytes compared with the current EC‐SERS. The strategy we present in this article enables the creation of portable and smart EC‐SERS analyzers for next‐generation biomedicines and catalysts.

## 2 Discussion and Results

**Figure 1:**
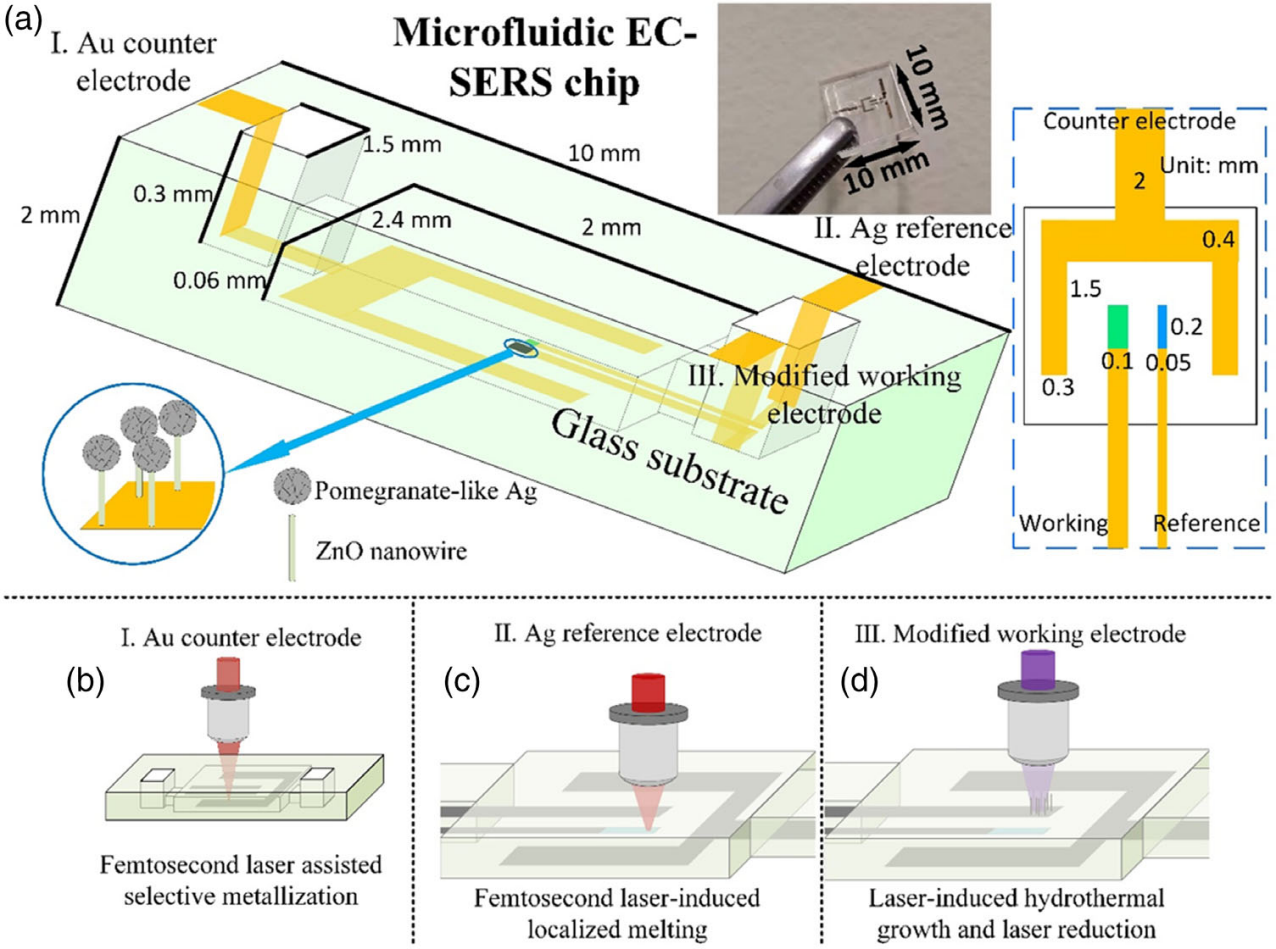
Configuration of microfluidic electrochemical surface‐enhanced Raman scattering (EC‐SERS) chip. a) Schematic of a three‐electrode system, in which Ag/ZnO nanostructures are used as the working electrode as shown in the magnified image at the lower left. An overall view photo of the microfluidic EC‐SERS chip is also inserted at the upper right. The dimension of the microfluidic EC‐SERS chip and each component is labeled in the inset. b–d) After fabrication of a 3D glass microfluidic chip by femtosecond laser‐assisted etching (FLAE), the gold counter electrode, silver reference electrode, and modified working electrode are fabricated by the ship‐in‐a‐bottle integration technique using successive processing consisting of fs laser‐assisted selective metallization, fs‐laser‐induced localized melting, and laser‐induced hydrothermal growth combined with laser reduction, respectively.

The configuration of the microfluidic EC‐SERS chip is shown in **Figure** **1**. The fabricated microfluidic EC‐SERS chip consists of a three‐electrode system, including a gold counter electrode, a silver reference electrode, and a modified working electrode. The electrodes were fabricated in a closed microfluidic chamber and electrically wired to the external glass surfaces through open micro‐reservoirs to connect them to the EC workstation using conductive copper tapes. The 3D glass microfluidic chip was first fabricated by FLAE, with details described in the Experimental Section. The dimensions and geometries of the microfluidic EC‐SERS chip are given in the Figure S1, Supporting information. The gold counter electrode (Figure 1b), silver reference electrode (Figure 1c), and modified working electrode were fabricated by the ship‐in‐a‐bottle integration technique using successive processing consisting of fs laser‐assisted selective metallization, fs‐laser‐induced localized melting, and laser‐induced hydrothermal growth combined with laser reduction, respectively.

### 2.1 fs Laser‐Assisted Selective Metallization in Microfluidic Channel

**Figure 2:**
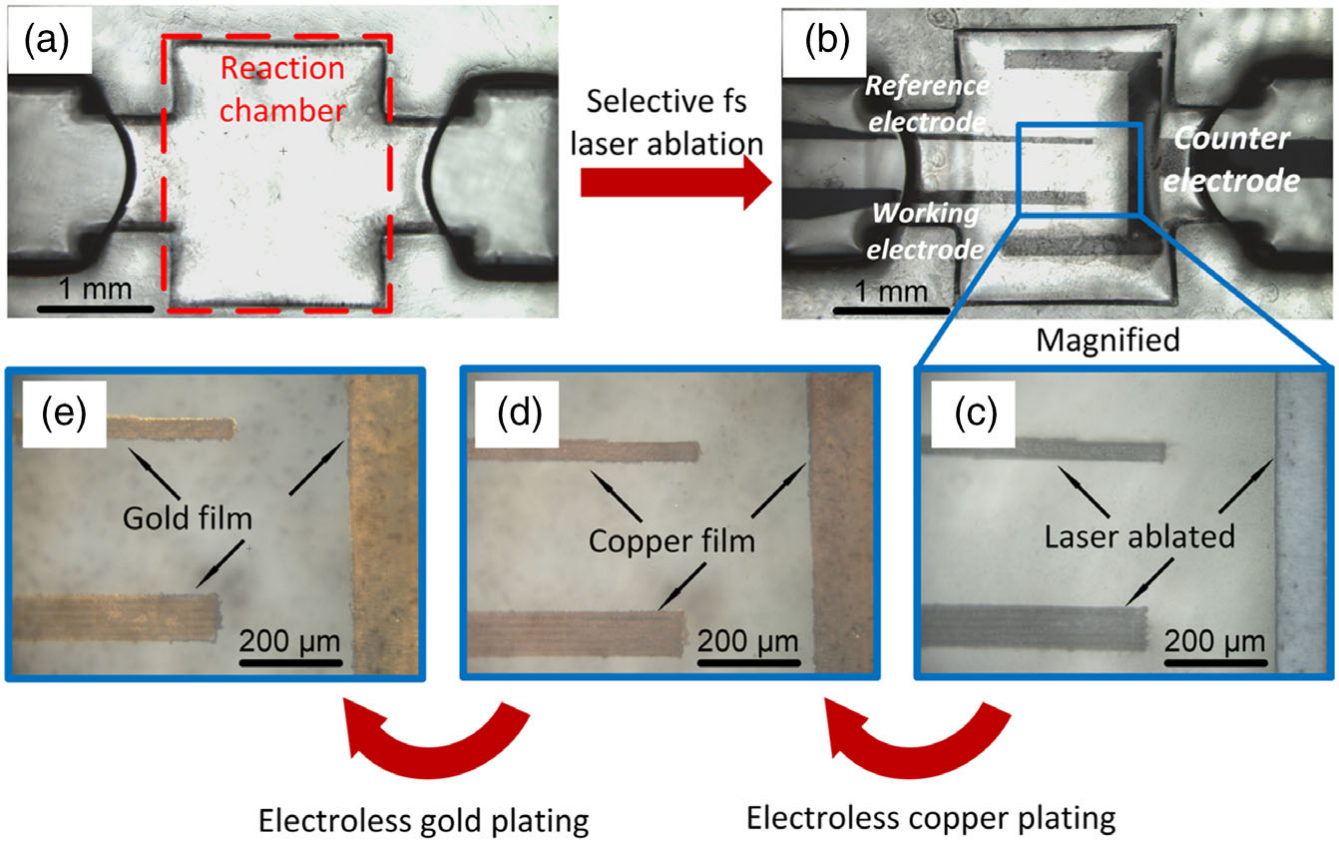
Three‐electrode system in microfluidic channel fabricated by ship‐in‐a‐bottle integration technique using advanced hybrid laser processing. a,b) Optical images of: a) after fabrication of 3D glass microfluidic chip by FLAE and b) after fs laser direct write ablation to selectively roughen the bottom surface of microchannel for subsequent fs laser‐assisted selective metallization. c) Magnified images of the blue rectangle region in (b) after ablation. Magnified images after copper deposition and gold deposition on the laser‐ablated regions by electroless metal plating are also shown in (d) and (e), respectively.

The glass microfluidic chip, shown in **Figure** **2**a, had a millimeter‐size closed chamber at the center, providing a space for EC‐SERS analysis. To create electrodes in the microfluidic chamber, fs laser direct write ablation was carried out to create designed structures on the bottom surface as shown in Figure 2b. As indicated by the arrows in Figure 2c, the fs‐laser‐ablated surfaces on which metal thin films were selectively deposited due to the anchor effect using electroless metal plating were somewhat roughened.^[^ ^12^ ^]^ First, a layer of copper film was formed by electroless copper plating, which was necessary to increase the bonding strength between the electrode and the glass substrate (Figure 2d). Then, a gold layer was deposited on the copper film by electroless gold plating. After laser fs laser‐assisted chemical etching, the glass surface became somewhat rough. Therefore, after the chemical etching, we employed 2nd annealing to smooth the etched surface. This thermal treatment improves the surface roughness with Ra better than 1 nm.^[^ ^13^ ^]^ Meanwhile, a part of the smoothed surface is again roughened by fs laser ablation for selective metallization by electroless plating. However, the roughness can be improved by the metallization process. In fact, the roughness of deposited metal film by the electroless plating is Ra = 0.4 μm,^[^ ^9^ ^]^ which is close to the flat film and doesn't affect the SERS signals. The U‐shaped gold/copper layer was used as the counter electrode, which surrounded the reference and working electrodes. Its size was much larger than the other two electrodes to achieve stable signals in electroanalysis.

### 2.2 Laser‐Induced Localized Melting of Silver Nanoparticles on Gold Substrate

**Figure 3:**
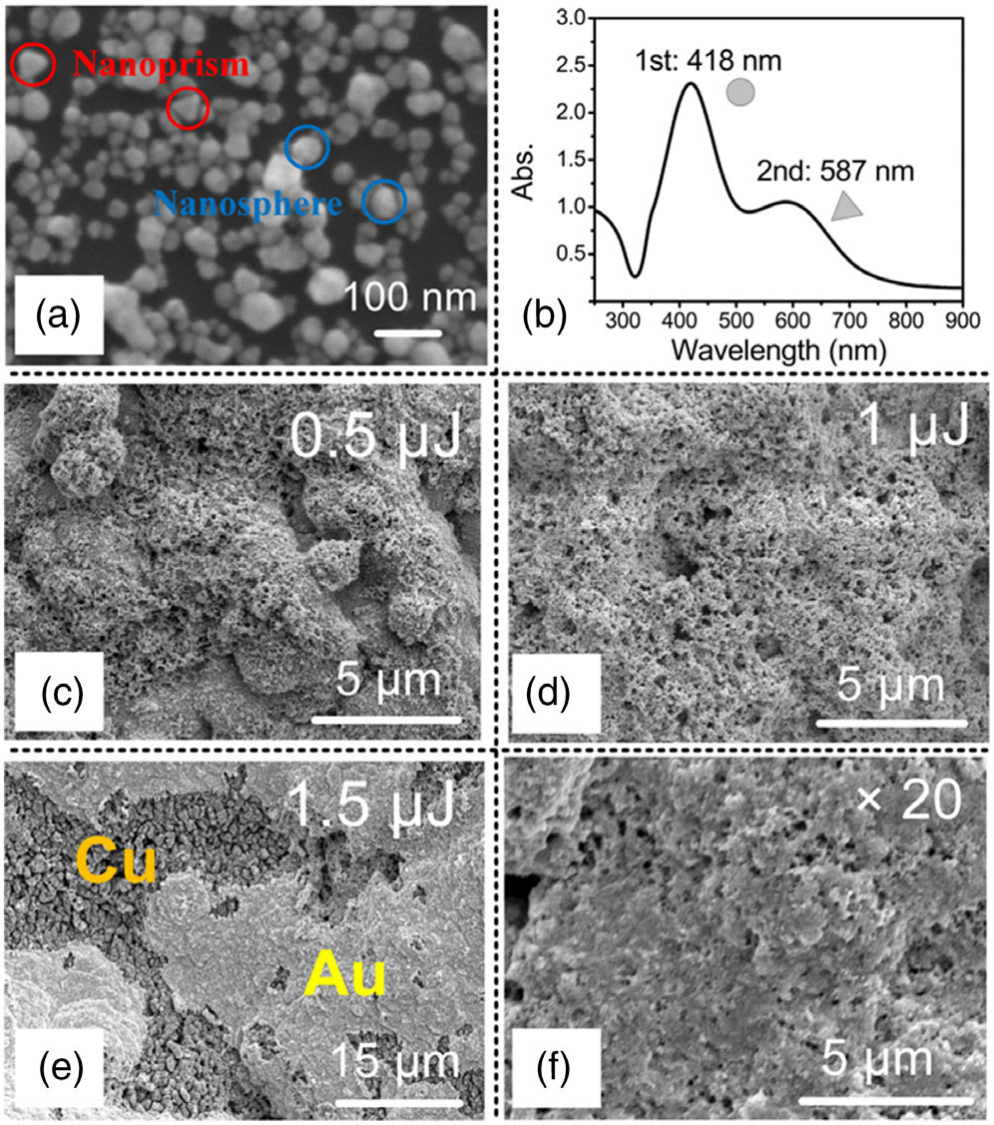
Femtosecond‐laser‐induced localized melting for the formation of a reference electrode. a) SEM image of silver nanoparticles synthesized by the wet‐chemical method. b) Absorbance spectrum of silver nanoparticle colloid. Absorption peaks at wavelengths centered at 418 and 587 nm are induced by the plasmon resonances of nanospheres and nanoprisms, respectively. c–e) SEM images of silver nanoparticles sintered on the gold film by fs‐laser‐induced localized melting at pulse energies of 0.5, 1, and 1.5 μJ, respectively. f) SEM image of gold film modified with silver for reference electrode by fs laser scanning 20 times at a pulse energy of 1 μJ.

The reference electrode was created by sintering silver nanoparticles using the method of fs‐laser‐induced localized melting on the gold/silver film formed by selective metallization. The sintering stabilizes the reference electrode so that repeatable signals can be achieved by ED analysis. Silver colloid was synthesized by a high‐yield wet‐chemical method which produced both nanospheres and nanoprisms.^[^ ^14^ ^]^ The morphology of the silver nanoparticles is shown in **Figure** **3**a. The mean size of the nanoparticles was 58.2 nm, which was measured by a zeta‐potential analyzer. The colloid had two absorption peaks at the central wavelengths of 418 and 587 nm, corresponding to nanospheres and nanoprisms, respectively, caused by dipole plasmon resonance (Figure 3b).^[^ ^15^ ^]^ During fs laser melting, a localized surface plasmon resonance (LSPR) will be induced to enhance the electric field near the surface of the nanoparticles (Figure S2, Supporting information). Although a nanosecond or even CW laser may be able to work for the sintering process, an fs laser should have the advantage of inducing minimal thermal damage to the underlying metal thin film due to the ultrashort pulse width. The enhanced electric field generates heat at the junction point of the silver nanoparticles and gold substrate, which sinters the silver nanoparticles onto the gold substrate because of the accumulation of heat during laser scanning.^[^ ^16^ ^]^ We employed nanoparticles instead of silver wires with micro/nanostructures to be sintered on the substrate, since the sintered nanoparticles were dense on the reference electrode, providing a stable environment. Whereas the wires may induce additional potentials due to the barriers at the contact interface, which will cause unreliable signals and decrease the efficiency of signal transmission. As shown in Figure 3c, when the pulse energy was set at around 0.5 μJ, the silver nanoparticles were sintered onto the gold substrate, although the sintered nanoparticles were unevenly distributed on the gold film. When the pulse energy was increased to 1 μJ, the gold film was mostly covered by silver nanoparticles and the silver composition ratio evaluated by energy‐dispersive spectroscopy (EDS) was significantly increased to 46 wt% as shown in **Table** **1**. However, it should be noted that the sintered silver nanoparticles formed a porous structure, which would degrade the stability of signals in electrochemical analysis. Further increase of the pulse energy to 1.5 μJ ablated the gold film and bared the underlayer (copper layer), so the pulse energy should be smaller than 1.5 μJ. To completely sinter the nanoparticles with no formation of porous structures, we scanned the fs laser beam 20 times at a pulse energy of 1 μJ (Figure 3f). Due to the increased opportunities for surface melting and joining of the silver nanoparticles, the sintered silver film became smoother with the increase in the number of laser scans. In the EDS, shown in Figure S3, Supporting information and Table 1, the silver composition ratio increased from 40.13 wt% (1 scan) to 60.49 wt% (5 scans), to 74.75 wt% (20 scans). Thus, the reference electrode fabricated by multiple laser scanning will provide reliable signals in EC‐SERS analysis.

**Table 1:** Relative ratios of silver, gold, and copper in wt% on the reference electrode measured by EDS for the samples prepared under different conditions

wt%	Silver	Gold	Copper
Gold film	0	68.65	21.84
Untreated	4.22	69.84	14.49
Electrode (0.5 μJ)	23.70	53.27	12.66
Electrode (1 μJ)	40.13	42.65	11.36
Electrode (5 scans)	60.49	24.55	11.36
Electrode (20 scans)	74.75	16.81	3.85

### 2.3 CW‐Laser‐Induced Hydrothermal Growth of Zinc Oxide Nanowires on Working Electrode

**Figure 4:**
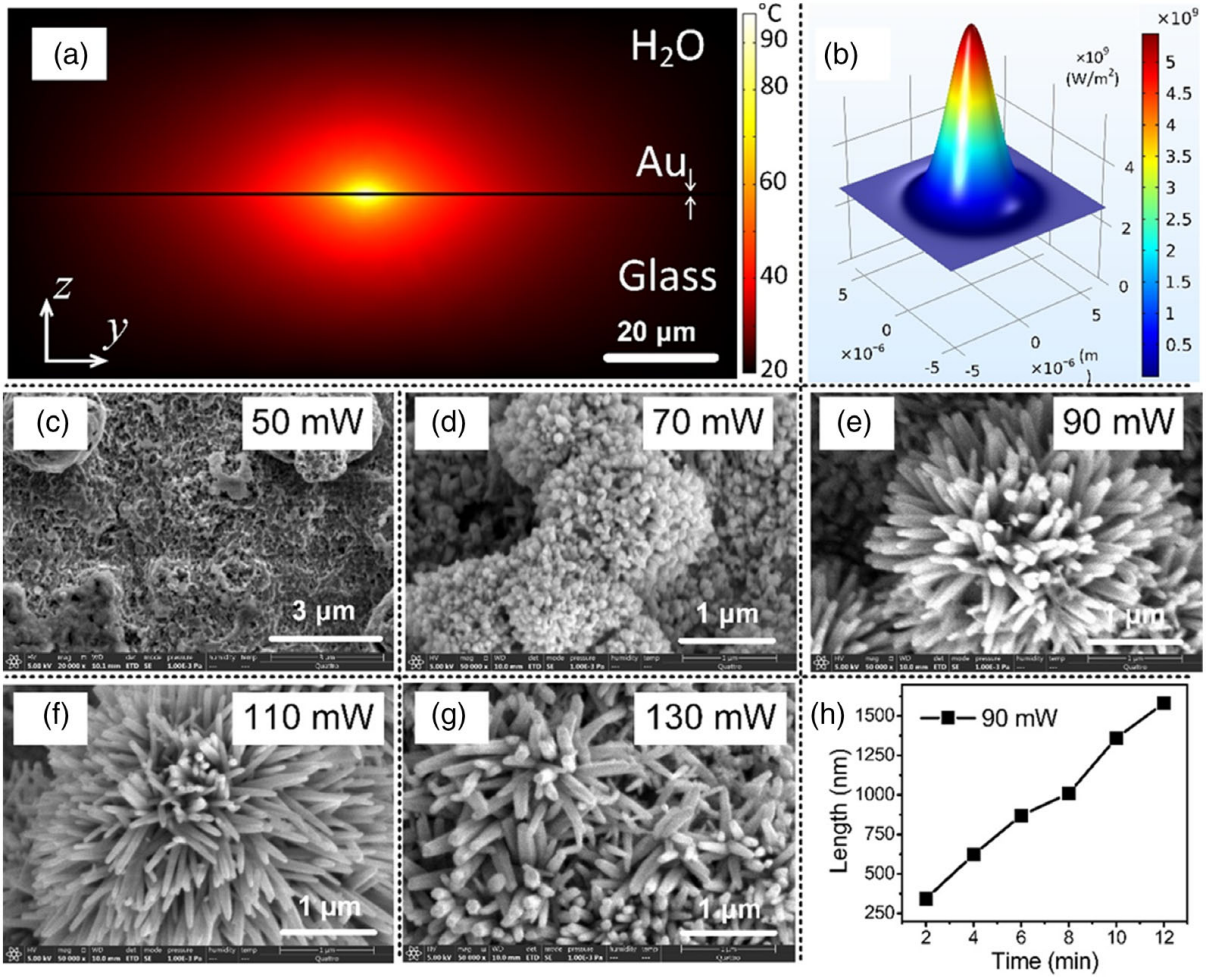
CW‐laser‐induced hydrothermal growth of zinc oxide nanowires on the working electrode. a) Temperature distribution in the *y–z* plane on thin gold film under CW laser irradiation. b) Spatial beam profile of laser beam used for the temperature simulation in (a). c–g) SEM images of zinc oxide nanowires synthesized on gold film by CW‐laser‐induced laser hydrothermal growth at laser powers of 50, 70, 90, 110, and 130 mW, respectively. The irradiation time was fixed at 10 min. h) Relationship of zinc oxide nanowire length with irradiation time at a laser power of 90 mW.

To induce the SERS effect, the working electrode must be modified; for this purpose, silver/zinc oxide hybrid nanostructures were created on the gold thin film formed by selective metallization. It has been demonstrated that silver/zinc oxide nanostructures are superior candidates for SERS applications owing to the involvement of both electric and chemical enhancement.^[^ ^17^ ^]^ We first synthesized zinc oxide nanowires by CW‐laser‐induced hydrothermal growth. Before synthesizing the zinc oxide nanowires, a layer of zinc oxide seeds was deposited on the gold thin film, which acted as the nuclei to facilitate zinc oxide growth along a specific crystal orientation for the creation of nanowires.^[^ ^18^ ^]^ The morphology of the zinc oxide seeds, which had average diameters below 10 μm, is shown in Figure S4a, Supporting information. The zinc precursor was pre‐heated to 50 °C using an infrared lamp and then quickly heated up to 60 °C by CW laser irradiation (**Figure** **4**a) to initiate the transition of zinc nitrate to zinc oxide due to the photothermal effect
(1)

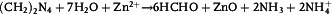

Since hexamethylenetetramine is split to form formaldehyde and ammonium in the reaction, zinc ions will combine with hydronium to form zinc hydroxide. Subsequently, zinc hydroxide creates multiple zinc oxide nuclei by the laser‐induced hydrothermal effect. Assisted by the polyethylenimine capping agent, the nucleated zinc oxide bonds to the zinc oxide seeds, resulting in oriented growth of zinc oxide at specific positions. The zinc oxide nanowires were measured by transmission electron microscopy (TEM) observation of a single nanowire and found to be about 200 nm in diameter with a length of >10 μm as shown in Figure S4b, Supporting information, and the cross‐section had a characteristic hexagonal shape (Figure S4c, Supporting information). Selected area electron diffraction revealed that the ZnO nanowires had a wurtzite structure with a single‐crystalline stem, as shown in Figure S4d, Supporting information. Since the growth of zinc oxide nanowires depends on the temperature of the precursor, we calculated the temperature increase on a gold film in the presence of a precursor under CW laser irradiation by finite element analysis. Assuming a Gaussian beam profile (Figure 4b), the highest temperature appears in the center of the laser spot. As shown in Figure 4a, the temperature distribution in the *y‐z* plane was simulated with laser scanning along the *y*‐axis and the temperature distribution in the *x‐y* plane in Figure S4f, Supporting information. The dynamic simulation of temperature is displayed in Video S1, Supporting information. The simulation showed that the temperature at the laser spot region maintained a value of 90 °C during laser scanning. Therefore, the zinc oxide nanowires continuously grew during laser irradiation, resulting in the formation of a uniform nanowire structure on the gold film. Figure S4e, Supporting information shows the growth of zinc oxide nanowires on a part of the gold film in a square region. Because of the charging effect, the zinc oxide region is brighter than the gold film region in the scanning electron microscopy (SEM) image. The homogeneous brightness of the zinc oxide region attests to the uniform growth of the nanowires on the gold film over a large area. Additionally, it is expected that the morphology of zinc oxide nanowires is governed by the laser power and irradiation time/scanning speed. In Figure 4c–g, we show the morphologies of nanowires synthesized over the laser power range of 50–130 mW. The results showed that the zinc oxide had a nanorod morphology at 70 mW, and became nanowires at 90 mW, nanotips at 110 mW, and spindle‐shaped structures at 130 mW with a reaction time of 10 min. We think that the resultant morphology will be determined by the reaction rate, and higher power provides a higher reaction rate. Figure 4h shows the dependence of the length of nanowires on the irradiation time at the optimal laser power of 90 mW. The length linearly increased as the irradiation time increased, as expected. It should be noted that for EC‐SERS analysis, 1 μm nanowires are long enough to provide good SERS performance because the excitation light cannot penetrate 3D structures of depth greater than 1 μm.^[^ ^19^ ^]^ Thus, the optimum conditions for the synthesis of zinc oxide nanowires by CW‐laser‐induced hydrothermal reaction were determined to be a laser power of 90 mW and an irradiation time of 10 min.

### 2.4 CW Laser Reduction of Silver Ions to Decorate the Zinc Oxide Nanowires

**Figure 5:**
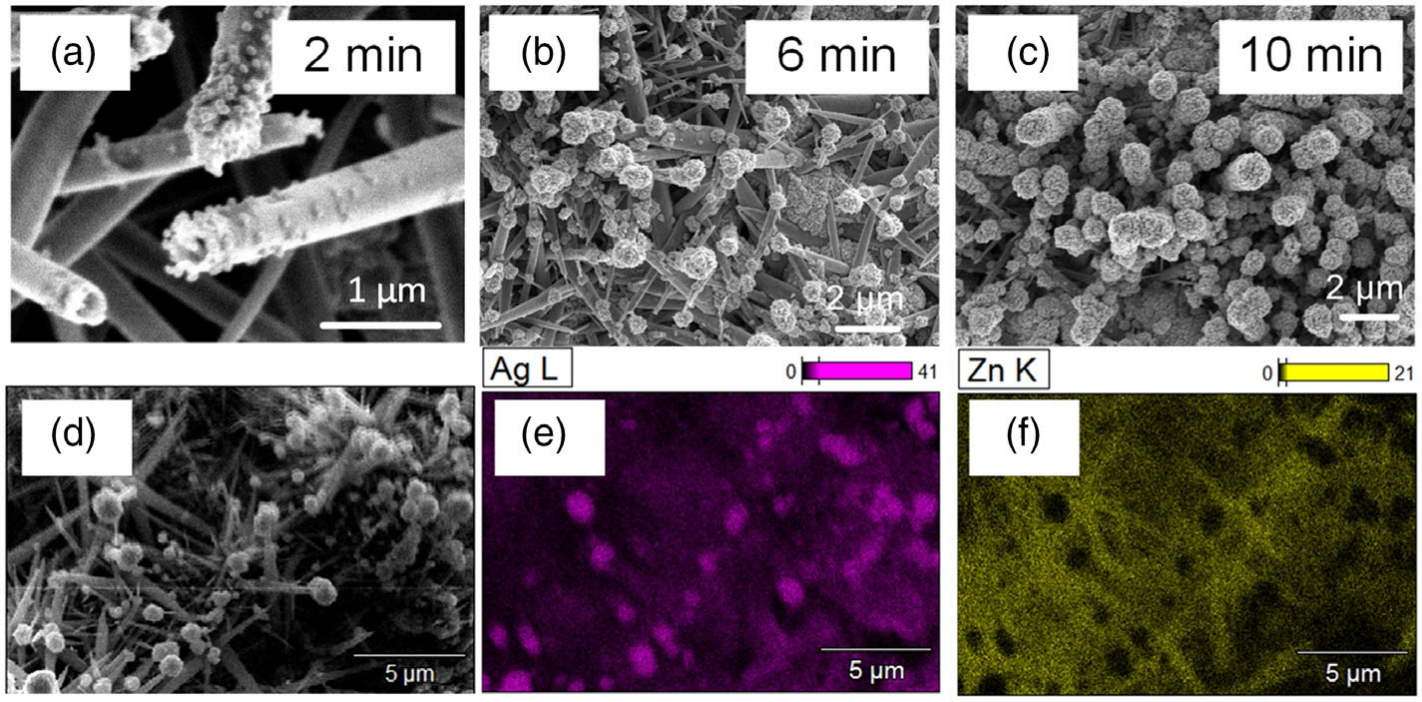
Decoration of zinc oxide nanowires with silver nanoparticles using CW laser reduction. a–c) CW laser reduction of silver ions at irradiation times of 2, 6, and 10 min, respectively. d) SEM image of silver/zinc oxide hybrid structures. e,f) EDS mapping of the SEM image in (d) with Ag L and Zn K signals, respectively.

Although zinc oxide itself can induce the SERS effect by charge transfer between the nanowires and analytes,^[^ ^20^ ^]^ decorating the nanowires with noble metal nanomaterials is preferable to achieve superior SERS performance.^[^ ^21^ ^]^ In our microfluidic EC‐SERS chip, we decorated the zinc oxide nanowires with silver nanoparticles. A typical CW laser reduction method was employed using silver nitrate and trisodium citrate as the precursors. The silver ions absorbed the photons and were reduced to silver atoms on irradiation with a 405 nm CW laser and then acted as silver nuclei. The silver nuclei induced LSPR to enhance the surface electric field and subsequently attracted the silver ions, leading to a continuous reduction process to form silver nanoparticles.^[^ ^22^ ^]^ In addition, the synthesized silver nanoparticles were capped by citrate, indicating the presence of a strongly negatively charged surface (zeta‐potential: −39.66 mW). The negative charges were accumulated at the silver nanoparticle surface to facilitate the growth of silver nanoparticles. Owing to Ostwald ripening and the aggregation of silver nanoparticles, the size of the particles grew with increasing laser irradiation time.^[^ ^23^ ^]^ **Figure** **5**a–c shows zinc oxide nanowires decorated with silver nanoparticles by a laser‐induced reduction for 2, 6, and 10 min, respectively. Since the wavelength we used was 405 nm, close to the absorbance of typical silver nanoparticles, the silver nanoparticles started growing as soon as the precursor was irradiated by the laser. At 2 min after the start of irradiation, the nanoparticles grew to ≈60 nm in diameter. Interestingly, we found that these nanoparticles were attached to the tips of zinc oxide nanowires and grew larger to cap the tips, until the zinc oxide nanowires were completely covered by silver nanoparticles at 10 min. The nanowires capped with nanoparticles were further observed and analyzed by SEM and EDS mapping, as shown in Figure 5d–f. The SEM image (Figure 5d) shows an interesting “candy apple” structure. The EDS mapping (Figure 5e (Ag L), and Figure 5f (Zn K)) illustrates that silver mapping shows a round shape, while the zinc mapping shows a wire shape with distinct boundaries, indicating that the apple part is silver and the stick part is zinc oxide in the “candy apple” structure.

**Figure 6:**
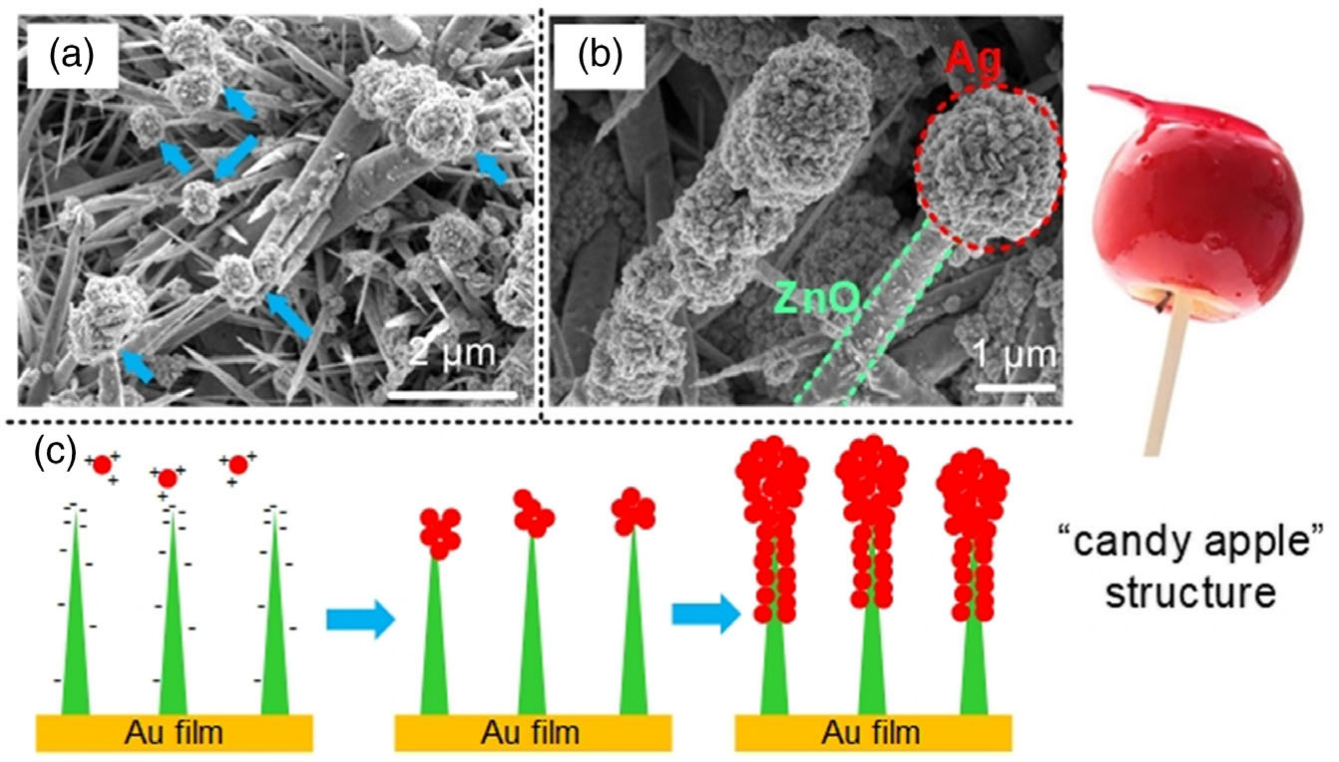
Images and schematic of the formation of “candy apple” structure. a) SEM image of “candy apple” structures. The laser irradiation time is 6 min. b) Magnified SEM image of “candy apple” structures. c) Schematic of growth dynamics of “candy apple” structure formed by CW laser irradiation.

We further investigated the “candy apple” structure by SEM, as shown in **Figure** **6**. The “apple” heads of the structures are indicated by blue arrows in Figure 6a. Although the zinc oxide nanowires were randomly distributed, the nanosized silver particles were capped on the tips nearly exclusively and few silver particles were deposited on the body of the nanowires. In the higher‐magnification SEM image (Figure 6b), two “candy apple” structures are presented. For the one at right, the diameters of the “candy” head composed of silver and the “stick” backbone of zinc oxide nanowire were measured as 1.5 μm and 200 nm, respectively. The “apple” head consisted of numerous nanoparticles to form a pomegranate‐like structure. It is worth mentioning that such a “candy apple” structure is very different from Ag/ZnO hybrid structures fabricated by other methods, such as chemical and ion sputtering methods.^[^ ^17^ ^]^ Such a novel “candy apple” structure would be more beneficial for SERS sensing.^[^ ^24^ ^]^ Specifically, the unique “candy apple” structure has several merits: 1) a highly enhanced localized electric field will be induced inside the aggregated structure with sub‐10 nm gaps formed in the pomegranate‐like silver structure (Figure S6b, Supporting information); 2) the high surface area of the “apple” due to the pomegranate‐like structure allowed larger numbers of analyte molecules to diffuse into proximity with hotspots; 3) zinc oxide nanowires surrounded by dense silver nanoparticles could improve the efficiency of charge transfer. With the further reduction, silver nanoparticles completely decorate the ZnO nanowire (left image in Figure 6b). We measured the Zeta potentials of silver nanoparticles and zinc oxide nanowires, revealing that the silver nanoparticles had a strong negative charge (−39.66 mV), while the zinc oxide nanowires had a positive charge (+14.54 mV). Therefore, in the laser reduction process, the nanoparticles can be adsorbed on the zinc oxide nanowire due to electrostatic interaction. Figure 6c schematically explains the formation of “candy apple” structures on the gold film. The zinc oxide nanowires synthesized by laser‐induced hydrothermal growth on gold film have higher positive charges on the tip of the nanowires than the body due to the tip concentration effect. Then, the negatively charged silver nanoparticles synthesized by laser reduction are preferentially adsorbed on the tips of the nanowires to form the “apple” head. With the further reduction, the nanoparticles gradually cover the whole surface of the zinc oxide nanowire due to Ostwald ripening. For EC‐SERS sensing, the “candy apple” structure is considered to be more effective than one with the zinc oxide nanowires covered entirely with silver nanoparticles, because the region of zinc oxide nanowire that is not covered with silver nanoparticles would generate hot electrons by light exposure, which transfer to the analyte molecules to enhance the intensity of the Raman signals.^[^ ^25^ ^]^ Thus, the “candy apple” structure was used for the following EC‐SERS analysis.

### 2.5 Evaluation of SERS Performances

**Figure 7:**
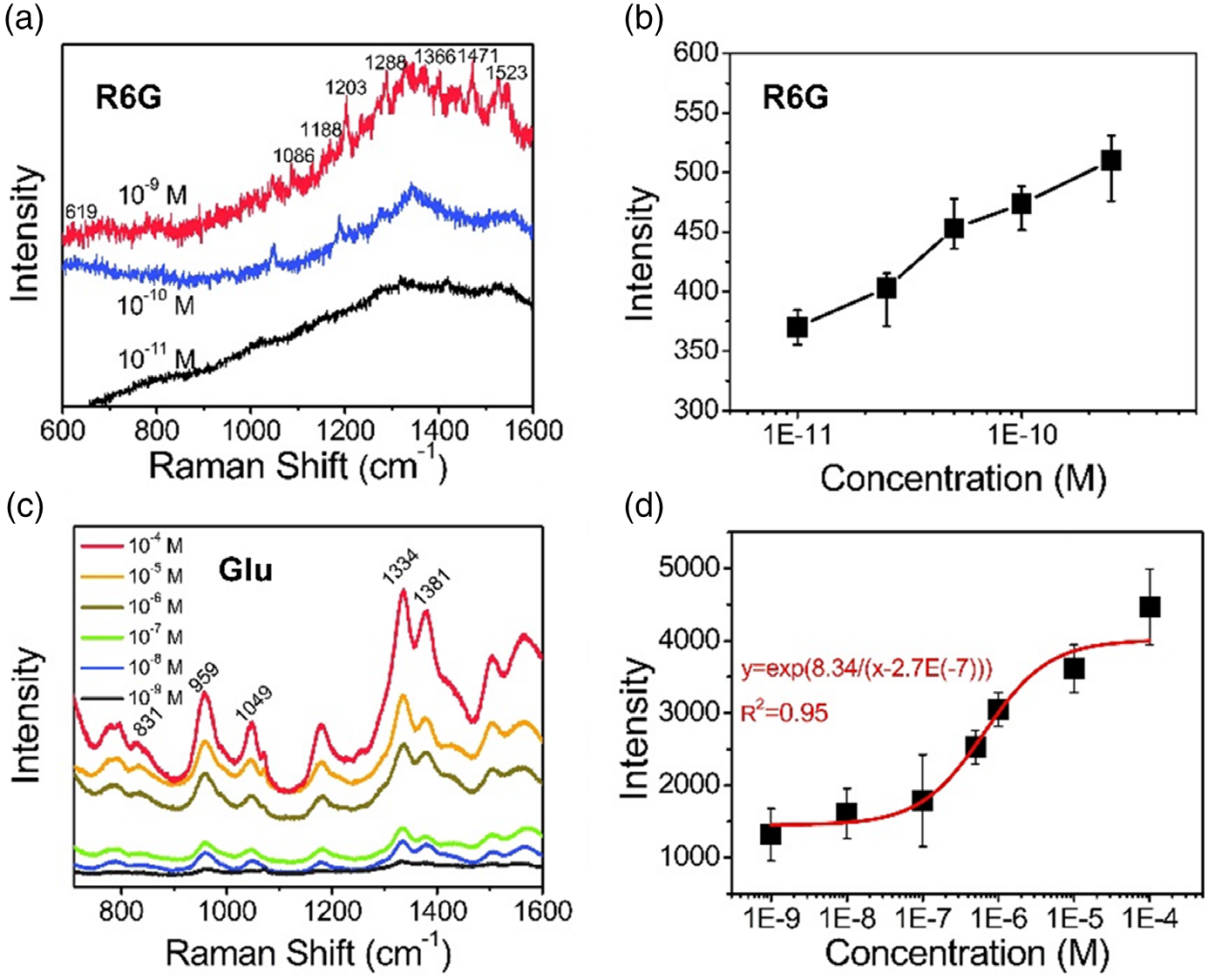
a) SERS spectra of R6G solutions with varied concentrations in the range of 10^−9^ to 10^−11^ m. b) Dependence of Raman peak intensity of R6G at 619 cm^−1^ on concentration. c) SERS spectra of Glu solutions with varied concentrations in the range of 10^−4^ to 10^−9^ m. d) Dependence of Raman peak intensity of Glu on concentration.

We investigated the SERS performance of fabricated chips using rhodamine 6G (R6G), which is a common dye molecule used to evaluate detection limits. As shown in **Figure** **7**a,b, the detection limit for R6G was evaluated as 90.16 pm, according to IUPAC (International Union of Pure and Applied Chemistry) definitions: 3.3 *σ*/*k*, where *σ* is the standard error of the *y*‐intercept in the regression line and *k* is the slope.^[^ ^26^ ^]^ The detection limit is one of the lowest ever achieved by regular SERS measurements. The relative standard deviation (RSD) was evaluated to be 10.4% by performing SERS measurements at 10 different positions on the EC‐SERS substrate. RSD will be further improved by the synthesis of a highly ordered “candy apple” structure, which will be realized by depositing a uniform laser absorbing layer on the metal film and focusing the laser from the backside of the sample. Such a low detection limit is attributed to the synergistic effects of charge transfer and LSPR, combined with photo‐induced enhancement in SERS.^[^ ^27^ ^]^

Additionally, the glutamate (Glu) was measured, which is one of the most ubiquitous neurotransmitters and a pathogenic factor for Alzheimer's disease.^[^ ^28^ ^]^ Since Glu requires high voltage to activate the electrochemical analysis when using the unmodified working electrode, we only present the SERS results of Glu to show the sensitivity in this study. Modifying the working electrode with an enzyme in the microfluidic EC‐SERS chip for Glu analysis will be our future work. The SERS results of Glu are shown in Figure 7c for different concentrations of Glu from 10^−4^ m to 10^−9^ m, showing the sensitivity of Glu is higher than the previous works.^[^ ^29^ ^]^ The Raman peak at 831 cm^−1^ was caused by the deformation modes of C—O and N—H Raman peaks at 1049 cm^−1^, while the peak at 1334 cm^−1^ was attributed to the deformation mode of amino groups at 959 and 1381 cm^−1^, which corresponded to the ionized form of Glu and symmetrical stretching of COO^−^ group, respectively.^[^ ^30^ ^]^ The Glu could be quantitatively detected with an exponential relationship between Raman intensity and concentration as *y* = exp(8.34/(*x*−2.7e(−7))) from 10^−4^ m to 10^−9^ m, as shown in Figure 7d.

### 2.6 EC‐SERS Analysis of Ascorbic Acid

**Figure 8:**
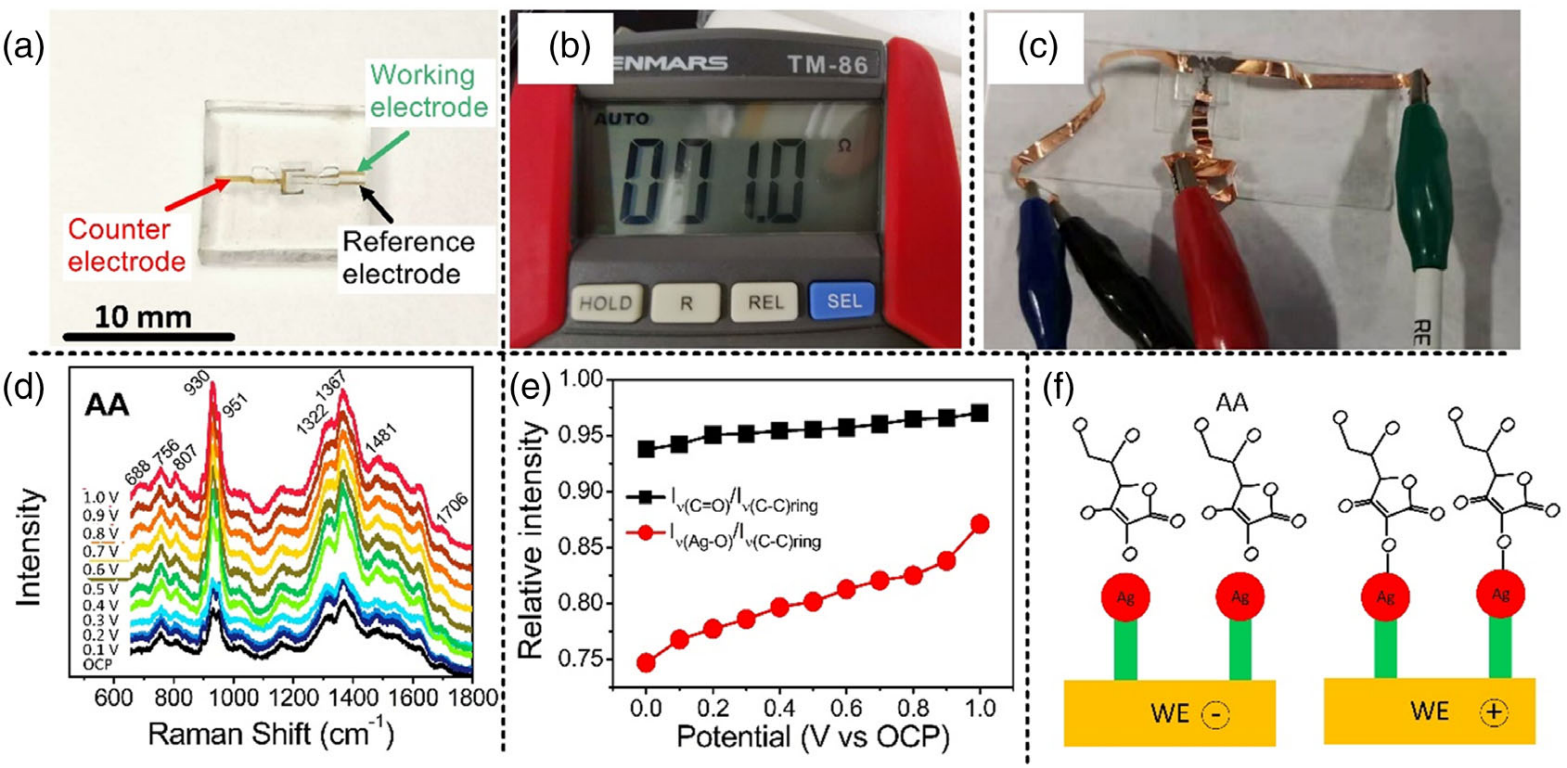
Analysis of neurotransmitters using microfluidic EC‐SERS chips. a) Overall view photograph of microfluidic EC‐SERS chip with a three‐electrode system. b) The resistivity of the electrode measured by a multimeter. c) EC‐SERS analysis scheme using microfluidic EC‐SERS chip. d) SERS spectra of ascorbic acid at the concentration of 10^−6^ m with different potentials applied to the working electrode. e) Variation of the intensity ratio of Raman vibrations at (C = O)_stretching_/(C—C)_ring stretching_ (blue line) and (Ag—O)_vibration_/(C—C)_ring_ (red line) with applied potential. f) Schematic of AA oxidation on the working electrode.

The fabricated microfluidic EC‐SERS chip was then used for the trace analysis of ascorbic acid (AA). AA is a natural antioxidant and participates in the metabolic processes of many creatures. **Figure** **8**a shows an overall view of the microfluidic EC‐SERS chip fabricated by hybrid laser processing. The size of the glass substrate was 10 × 10 × 2 mm^3^ (fingernail size), on which the three‐electrode system was fabricated within an area of 2 × 2.4 mm^2^. It should be noted that the typical size of the conventional screen‐printed EC‐SERS electrode chip is 30 × 8 mm^2^ and a palm‐sized container is required for the integration of the electrodes.^[^ ^31^ ^]^ Thus, the ship‐in‐a‐bottle integration using hybrid laser processing can reduce the chip size to less than 1/50 that of SPEs. As another important feature, the volume of the reaction chamber for a conventional screen‐printed EC electrode is ≈100 mL, whereas that for our microfluidic EC‐SERS chip is only ≈3 μL, which is several orders of magnitude smaller than the conventional SPE‐based EC‐SERS device. The counter electrode, reference electrode, and working electrode in the three‐electrode system, which were created in the glass microfluidic chamber and electrically connected to the external surface of the glass substrate, are labeled in the photograph. The resistivity of the gold thin film connecting the internal (in microchamber) to the external (on glass) surface formed by the fs laser‐assisted selective metallization was as low as 1 Ω (Figure 8b). This illustrated that a conductive metal film was continuously coated on the vertical sidewalls of the glass microchannel by our proposed method.^[^ ^32^ ^]^ The conductivity of the electrodes was measured to be ≈2.2 × 10^7^ Sm^−1^. Although the conductivity was about two times lower than bulk gold, the conductivity was sufficiently low for EC‐SERS applications. The microfluidic EC‐SERS chip was connected to an EC workstation using copper conductive tape, as shown in Figure 8c, and then the Raman signals were collected using a conventional Raman spectrometer to analyze the neurotransmitters by EC‐SERS.

Figure 8d–f shows the results of AA analysis and the schematic of AA oxidation on the working electrode when applying a positive potential. 10^−6^ m AA in a buffer solution with a total volume of ≈10 μL was injected into the microfluidic chip. The required volume was much smaller than that of a conventional EC device (≈5 mL)^[^ ^33^ ^]^ and SPE (≈100 mL).^[^ ^34^ ^]^ The details of the EC‐SERS measurements are described in the Methods section. We applied positive potentials to the working electrode from the open circuit potential to +1 V and then acquired SERS spectra at each potential as shown in Figure 8d. With the increase in the potential to +1 V, the Raman scattering intensity of AA increased, which means that the AA molecules in the solution moved towards the working electrode to gather on it. The increase in Raman scattering intensity with potential is also due to the charge transfer between the AA and silver/zinc oxide nanostructures, since high potential facilitates the charge transfer to achieve stronger SERS intensity. We found that the Raman intensity was dramatically increased when a potential of +0.4 V was applied. This dramatic increase can be attributed to the oxidation of AA to bond with the working electrode. Thus, the AA is tightly attached to the silver surface, leading to a remarkable increase in SERS intensity. The oxidation behavior was investigated by *I–V* analysis with differential pulse voltammetry (Figure S6a, Supporting Information), showing a great increase in the oxidation current at potentials above +0.2 V; the maximum oxidation current occurred at +0.5 V with a drastic jump.^[^ ^35^ ^]^ More significantly, the bonding of AA on the working electrode gave rise to a new Raman peak at 688 cm^−1^, which can be assigned to the vibration of Ag‐O. In addition, the Raman scattering intensity at 688 cm^−1^ increased as the potential increased, indicating that the number of AA molecules bonded on the working electrode increased due to the increase in oxidation at a higher potential. We calculated the relative intensities (ratios) of two specific Raman peaks at (C=O)_stretching_/(C—C)_ring‐stretching_ and (Ag—O)_vibration_/(C—C)_ring‐stretching_ as a function of applied positive potential (Figure 7e). Considering the chemical structure of AA molecules, the number of C=O and Ag—O bonds increases because of AA oxidation and AA—silver bonding, while the number of C—C bonds is unchanged. Therefore, the two ratios increase from 93.8% to 97.0% and 74.7% to 87.1% on increasing the positive potential applied to the working electrode, respectively. In addition, since Ag—O bonds are induced by the oxidation of AA on the silver surface, the ratio of (Ag—O)_vibration_/(C—C)_ring‐stretching_ is more prominent than the ratio of (C=O)_stretching_/(C—C)_ring‐stretching_. As illustrated in Figure 8f, the redox reaction of AA occurs on the working electrode as follows

(2) $$2{\text{Ag}}^{+}+C_{6}H_{8}O_{6}⇌2Ag+C_{6}H_{6}O_{6}+2H^{+}.$$

On applying a positive potential (>0.2 V), AA attaches to the silver nanoparticles and undergoes oxidation, and the Raman scattering intensity of the Ag—O bonds formed becomes stronger at higher potentials. In contrast, this reaction can be reversed by applying a negative potential to the working electrode to detach AA from the silver nanoparticles. Thus, the microfluidic EC‐SERS chip should be reusable after applying a negative voltage as a cleaning process. As a demonstration, after AA measurements, the chip was cleaned by applying a negative potential in potassium chloride solution with stirring, as schematically shown in Figure S7a, Supporting Information. Then, the cleaned chip was tested again, which showed that no Raman signals were detected, except for the background noise (Figure S7b, Supporting Information). However, when reused more than three times, the performance of the chip began to deteriorate, which meant that AA could not be completely eliminated by the cleaning process (Figure S7c, Supporting Information). Furthermore, a vitamin necessary for the biosynthesis of some neurotransmitters was analyzed by the fabricated EC‐SERS chip. Importantly, the volume of vitamin solution required for EC‐SERS analysis using the microfluidic EC‐SERS chip is tens of times smaller than other methods such as modified SPE chips and regular EC electrodes (Table S1, Supporting Information).^[^ ^36^ ^]^ Consequently, the EC‐SERS chip we fabricated was ultraminiaturized to 1/50 the size of a typical SPE device. For the EC‐SERS analysis of neurotransmitters (Glu), the three electrodes should be modified with an enzyme such as glutamate oxidase. Three electrodes modified with the enzyme such as glutamate oxidase have the ability to provide enough electrons and then oxidize Glu to be ketoglutarate, even if the low potential is applied to the working electrode.^[^ ^37^ ^]^ Thus, it is reasonable to expect that our microfluidic EC‐SERS chip is applicable for the analysis of Glu and other neurotransmitters.

Our ingenious microfluidic EC‐SERS chip fabricated by ship‐in‐a‐bottle integration based on hybrid laser processing significantly outperforms conventional devices not only in terms of analyte volume but also portability. Portability is a key factor for point‐of‐care testing, while the minimized analyte volume benefits patients by reducing the pain associated with testing. Thus, our technique holds promise for the fabrication of next‐generation EC‐SERS analyzers for biomedicines and catalysts.

## 3 Conclusions

We fabricated a microfluidic EC‐SERS chip by ship‐in‐a‐bottle integration using hybrid laser processing for the analysis of neurotransmitters. Compared to the conventional EC‐SERS devices fabricated based on screen‐printed electrodes, the device size was miniaturized to an extraordinary extent, from palm size to finger‐nail size. Consequently, the required analyte volume was able to be reduced to sub‐10 μL levels, which is several orders smaller than that required for conventional devices. The advanced hybrid laser processing we adopted consisted of five consecutive laser processing steps including FLAE, fs laser‐assisted selective metallization, fs‐laser‐induced localized melting, CW‐laser‐induced hydrothermal growth, and CW laser reduction. In the glass microfluidic chip fabricated by FLAE, a three‐electrode system consisting of a gold counter electrode, silver reference electrode, and modified working electrode was created by the ship‐in‐a‐bottle integration technique. The reference electrode was created by fs‐laser‐induced localized melting of silver nanoparticles on a gold thin film. The working electrode was further formed by CW‐laser‐induced hydrothermal growth of zinc oxide nanowires and CW laser reduction to decorate the grown zinc oxide nanowires with silver nanoparticles. Interestingly, a “candy apple” structure was formed on the working electrode due to electrostatic interactions, in which silver nanoparticles formed an “apple head” on a zinc oxide nanowire serving as the stick. To assess the fabricated microfluidic EC‐SERS chip, we elucidated the redox reaction of 1 μm AA in a sub‐10 μL volume on the working electrode by measuring EC‐SERS spectra at different applied potentials, demonstrating the superior performance of the chip for EC‐SERS analysis. Furthermore, we revealed that the chip could be repeatedly used after proper cleaning with an applied negative potential. The results show that the ship‐in‐a‐bottle integration technique is a versatile method to process otherwise intractable materials. The fabricated functionalized microfluidic chip can be used for the next generation of EC‐SERS analyzers with a very small footprint for early disease diagnosis and point‐of‐care testing.

## 4 Experimental Section

4.1

4.1.1

##### Glass Microfluidic Channel Fabricated by Femtosecond‐Laser‐Assisted Chemical Etching

The photosensitive glass substrates (FoturanII) with a size of 10 × 10 × 2 mm^3^ were purchased from Schott. To create a 3D microfluidic channel, an fs laser (CB5‐06, Carbide) with a wavelength of 1030 nm, a repetition rate of 200 kHz, and a pulse width of 223 fs was employed. The pulse energy was set at 0.15 μJ and the laser beam was focused by a 20× objective lens (M Plan Apo NIR, Mitutoyo) with a numerical aperture of 0.4. The setup of the laser system is shown in Figure S8, Supporting Information. A 3D motorized translation stage was used for laser scanning according to the designed 3D structures. After fs laser scanning, the glass substrate was successively treated by annealing for the development of modified regions, chemical etching for the formation of 3D microfluidic structures, and post‐annealing processes for smoothing the etched surfaces with average roughness better than 1 nm to create 3D microfluidic channels inside the glass chip. The details can be found in Figure S9a, Supporting Information and previous works.^[^ ^9^, ^10^ ^]^

##### fs Laser‐Assisted Selective Metallization

The electrodes were created in the microfluidic channel by electroless plating of the gold film. To selectively deposit a gold film on the bottom surface of the channel, the channel was filled with water and then the target regions were ablated by an fs laser. In laser ablation, the pulse energy was set at 1.5 μJ and other conditions were the same as described in Section 4.1. The geometry of the fs laser‐ablated regions in the channel is labeled in Figure S9b, Supporting Information. After laser ablation, the glass substrate was ultrasonically cleaned in 0.1 m HCl and distilled water for 10 min each to eliminate contamination in the laser‐ablated regions. An electroless copper plating solution (C‐200LT, Kojundo Chemical Laboratory Co., Ltd.) was first used to deposit a copper film on the laser‐ablated regions to ensure the adhesion of subsequent metal thin films on the glass surfaces. The plating was carried out at a temperature of 50 °C for 3 h using a water bath with a stirring function. Next, a gold film was coated on the deposited copper film using an electroless gold plating solution (K‐24N, Kojundo Chemical Laboratory Co., Ltd.). The plating was performed at a temperature of 70 °C for 1 h in the same water bath. Finally, the glass substrate was successively rinsed with distilled water and acetone.

##### Laser‐Induced Localized Melting of Silver Nanoparticles

The silver nanoparticles were synthesized by a wet‐chemical method. Briefly, 15 × g glucose and 5 × g poly(vinylpyrrolidone) (PVP, K‐30) were dissolved in 300 mL deionized water. The pH of the solution was adjusted to 11 using sodium hydroxide. Then, the solution was heated to 70 °C under stirring and 100 mL (0.35 m) silver solution was added to the heated solution through a dropper using a micropump. After the reaction, the silver nanoparticle solution was cleaned several times in ethanol to remove PVP using a centrifuge at 5000 rpm, which concentrated the solution to 20 mL. Next, 8 μL of the concentrated solution was injected into the microfluidic channel for fs laser localized melting. For this process, a 100 kHz 1030 nm fs laser was focused by a 5× objective lens (M Plan Apo NIR, Mitutoyo) onto the gold film in the microfluidic channel. The laser scanning speed was set at 100 μm s^−1^ and the pulse energy was 50 nJ, as shown in Figure S9c, Supporting Information.

##### Modification of Working Electrode for SERS Sensing

The growth of zinc oxide nanowires was carried out with reference to ref. [38]. Briefly, the zinc oxide seeds were synthesized using 30 × 10^−3^ m NaOH and 10 × 10^−3^ m zinc acetate dehydrate in ethanol. Then, the zinc oxide seeds were deposited onto the gold film as nuclei for the growth of zinc oxide nanowires. In the growth of zinc oxide nanowires, 25 × 10^−3^ m of zinc nitrate hexahydrate and 15 × 10^−3^ m hexamethylenetetramine were mixed, and then ≈6 × 10^−3^ m polyethylenimine was added into the solution. For the laser‐induced hydrothermal growth, a 405 nm CW laser (Cobalt 06‐01) was used to scan the substrate at a speed of 60 μm min^−1^, during which the solution was kept at 50 °C using an infrared lamp. The laser was focused by a 10× objective lens (M Plan Apo, Mitutoyo). The scanned area was 100 × 100 μm^2^, as shown in Figure S1c, Supporting Information (green region). For decoration of the zinc oxide nanowires, silver nanoparticles were synthesized by laser reduction. The precursor solution was prepared with 10 × 10^−3^ m silver nitrate and 0.5 × 10^−3^ m trisodium citrate dihydrate, which was introduced into the microfluidic channel. The 405 nm CW laser also used for the laser‐induced growth was focused by a 2× objective lens (M Plan Apo, Mitutoyo) as shown in Figure S9d(2), Supporting Information. After laser irradiation, the sample was rinsed several times with deionized water.

##### Characterization

High‐resolution images of the nanostructured metal films were obtained using field‐emission scanning electron microscopy (Quattro, Thermo Scientific), and optical images of the microchannels and metal films were recorded with a 3D profiler (Zeta‐20, KLA). The Raman spectra were collected by a Raman spectrometer (NRS4500, JASCO) equipped with a microscope. The excitation power and exposure time were 2.8 mW and 10 s, respectively. The excitation laser beam with a wavelength of 633 nm was focused on the working electrode using a 50× objective lens with a numerical aperture of 0.5. EC spectra were acquired by an EC measurement system (HZ‐7000, Hokuto Denko).

##### Simulation

The electric field and heat transfer simulations were performed with COMSOL Multiphysics. The necessary parameters for the materials were taken from the data in ref. [39]. A plane wave was incident on the sample at normal incidence. For the simulation of heat transfer, fluid dynamics with laminar flow in conjugation with the heat transfer in fluids were adopted. The boundary conditions consisted of a boundary heat source at the gold surface to simulate laser heating. The temperature at other contour boundaries was set at room temperature.

## Conflict of Interest

The authors declare no conflict of interest.

## Supporting information

Supplementary Material

## Data Availability

The data that support the findings of this study are available from the corresponding author upon reasonable request.
