# Supplementary material for: Ultraminiaturized Microfluidic Electrochemical Surface‐Enhanced Raman Scattering Chip for Analysis of Neurotransmitters Fabricated by Ship‐in‐a‐Bottle Integration
Source: Small Sci. 2023 Jan 29;3(3):2200093. doi: 10.1002/smsc.202200093 (PMC11935879; doi:10.1002/smsc.202200093)
Supplement: Supplementary file 1 — Supplementary Material [file SMSC-3-2200093-s001.zip › smsc202200093-sup-0001-SuppData-S1.pdf]

Supplementary information

Ultraminiaturized microfluidic electrochemical surface-enhanced Raman scattering chip for analysis of neurotransmitters fabricated by ship-in-a-bottle integration

Shi Bai,[1,2] Ying Ma,[3] Kotaro Obata,[1] Koji Sugioka[1]*

1. Advanced Laser Processing Research Team, RIKEN Center for Advanced Photonics, 2-1 Hirosawa, Wako, Saitama 351-0198, Japan
2. School of Material Science and Engineering, Hebei University of Science and Technology, Shijiazhuang 050018, China
3. Academy of artificial intelligence, Beijing institute of petrochemical technology, No.19 North Qingyuan Road, Daxing District 102617, Beijing, China,
* Correspondence: ksugioka@riken.jp

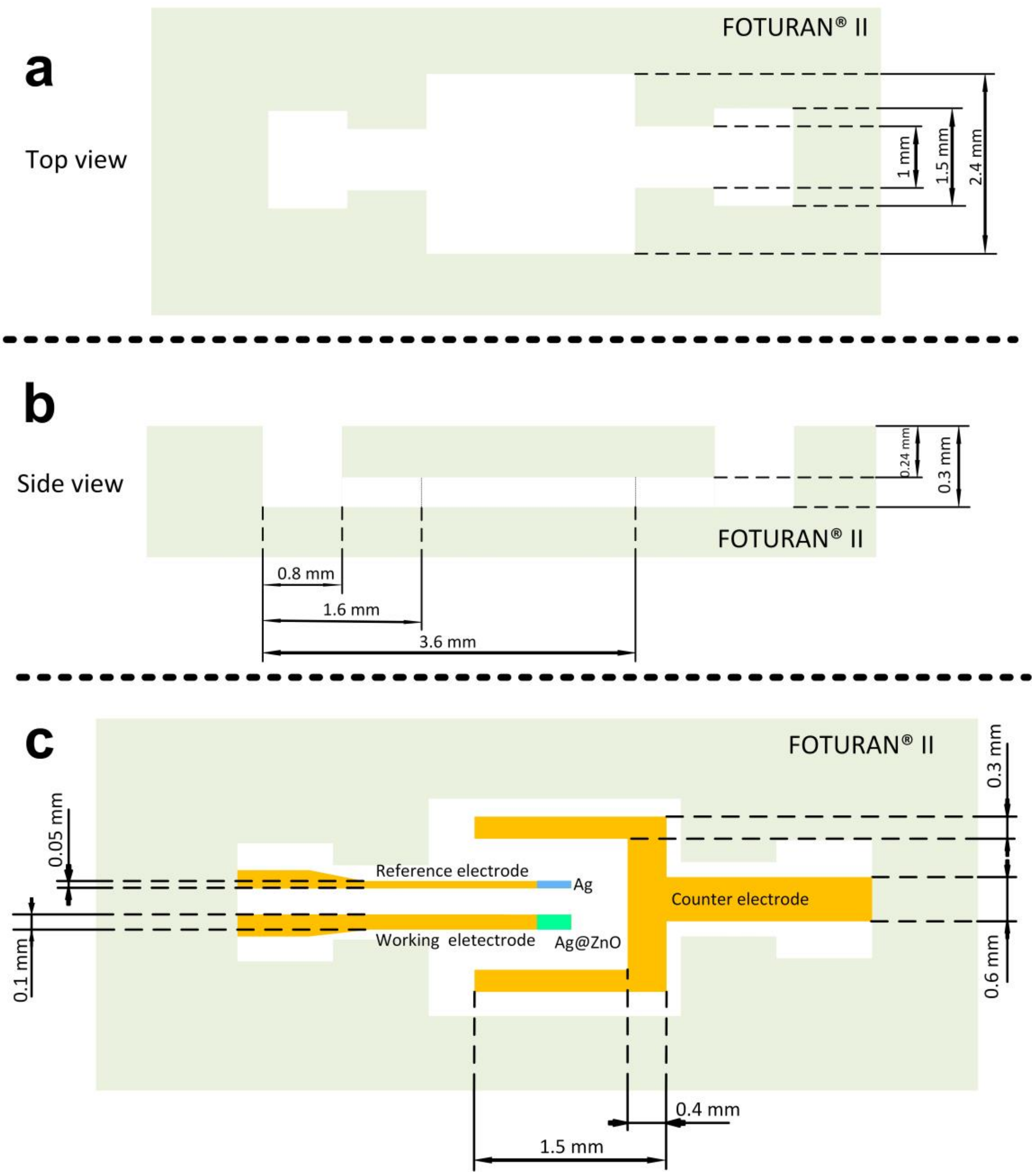

Figure S1. The dimensions and geometries of microfluidic EC-SERS chip. (a) – (b) Top and side views of the designed glass microfluidic chip. (c) The dimensions of three-electrode system in glass microchamber.

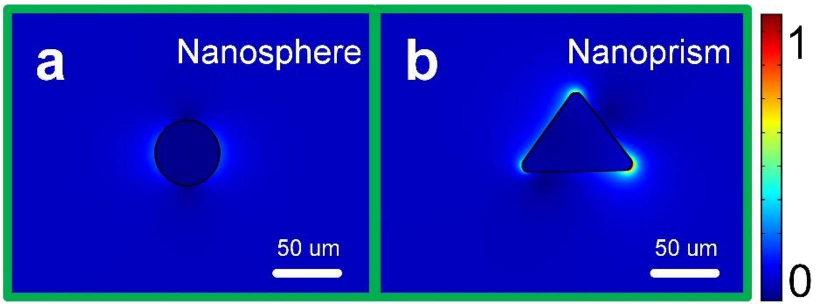

Figure S2. Electric field enhancement of incident light by (a) nanosphere and (b) nanoprism synthesized by wet-chemical method.

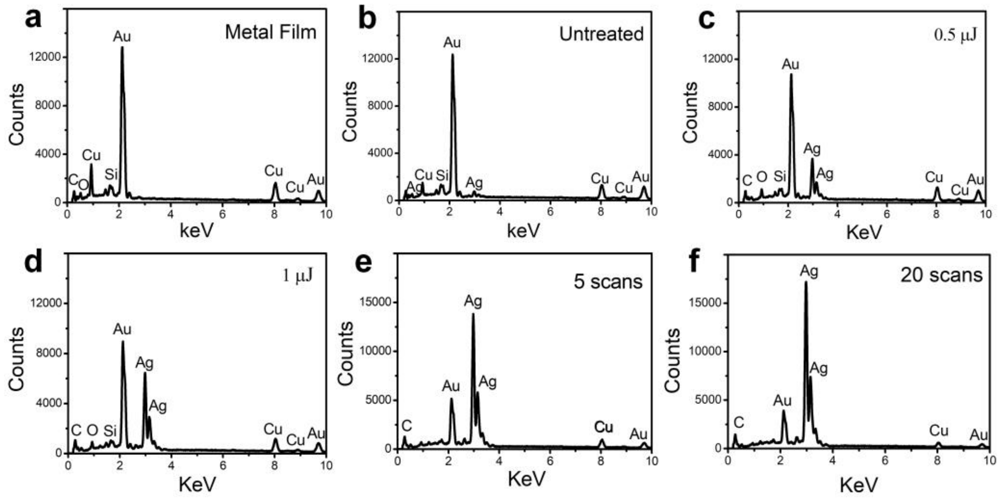

Figure S3. Energy dispersive Spectroscopy (EDS) analysis of the reference electrode. (a) EDS spectrum of electrode after gold coating. (b) EDS spectrum of silver nanoparticle deposited gold film. (c), (d) EDS spectra of silver sintered gold film at the pulse energy of 0.5 and 1 μJ, respectively. (e), (f) EDS spectra of silver sintered gold films at the pulse energy of 1 μJ after 5 and 20 laser scans, respectively.

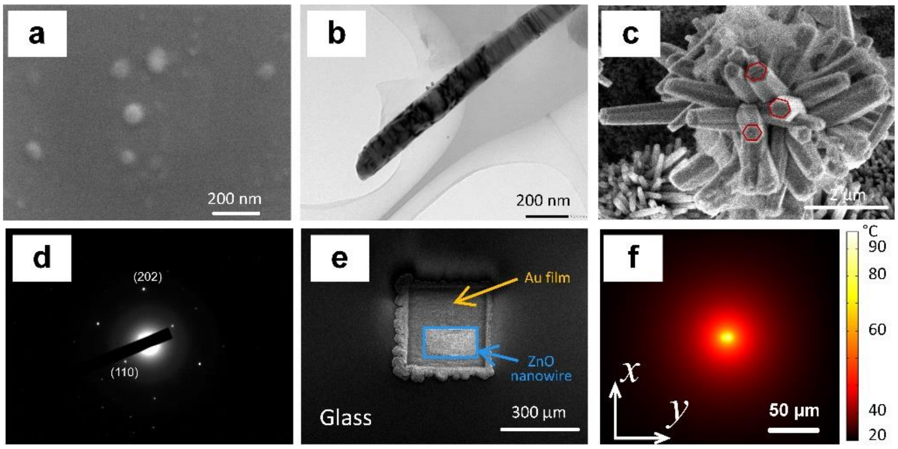

Figure S4. (a) SEM image of zinc oxide seeds. (b) TEM image of single zinc oxide nanowire. (c) The synthesized zinc oxide nanowire, showing a hexagonal shape at cross-section. (d) The pattern of selected area electron diffraction of zinc oxide, demonstrating the single crystal structure. (e) Zinc oxide nanowires grown on gold film. The zinc oxide nanowire and gold film are labeled in the image. (f) Temperature distribution in *x*-*y* plane on thin gold film under CW laser irradiation.

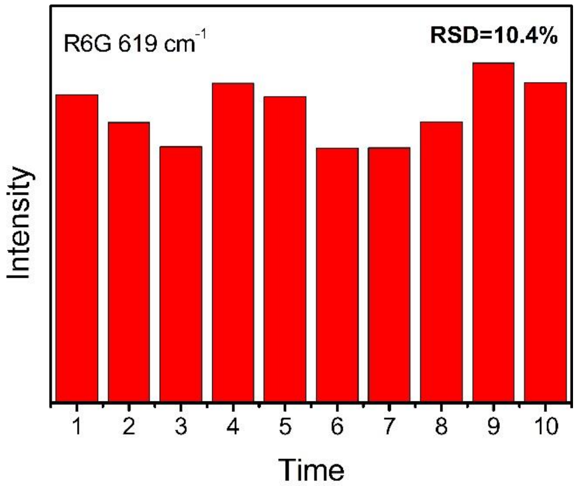

Figure S5. The relative standard deviation of SERS signals of R6G at 619 cm^{-1} measured on 10 arbitrary locations.

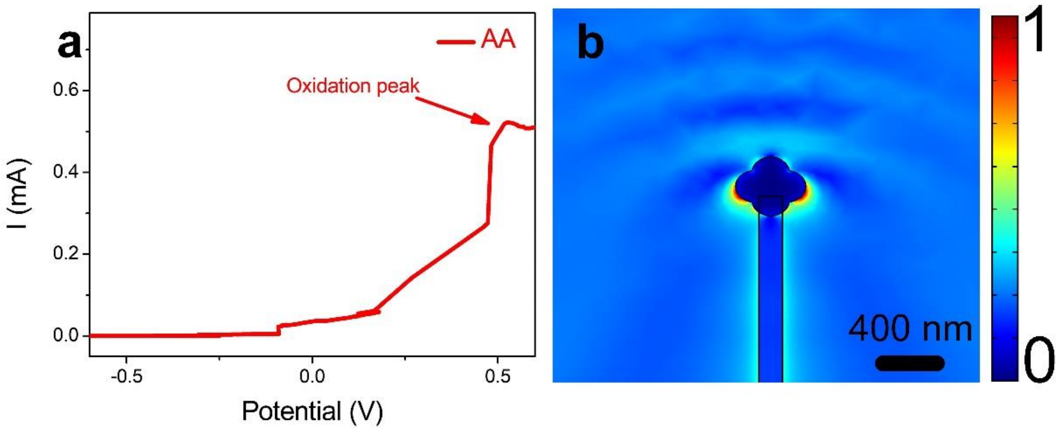

Figure S6. (a) Differential pulse voltammetry of ascorbic acid using microfluidic EC-SERS chip. The concentration of AA is 1 μM. (b) Electric field distribution enhanced by a "candy apply" structure with a pomegranate-like structure by light excitation. The dimension of pomegranate-like structure was measured from an SEM image (Figure 5b), showing a size of ~ 500 nm. Zinc oxide observed from a TEM image has a diameter of 200 nm and a length of 10 μm (Figure S4 b).

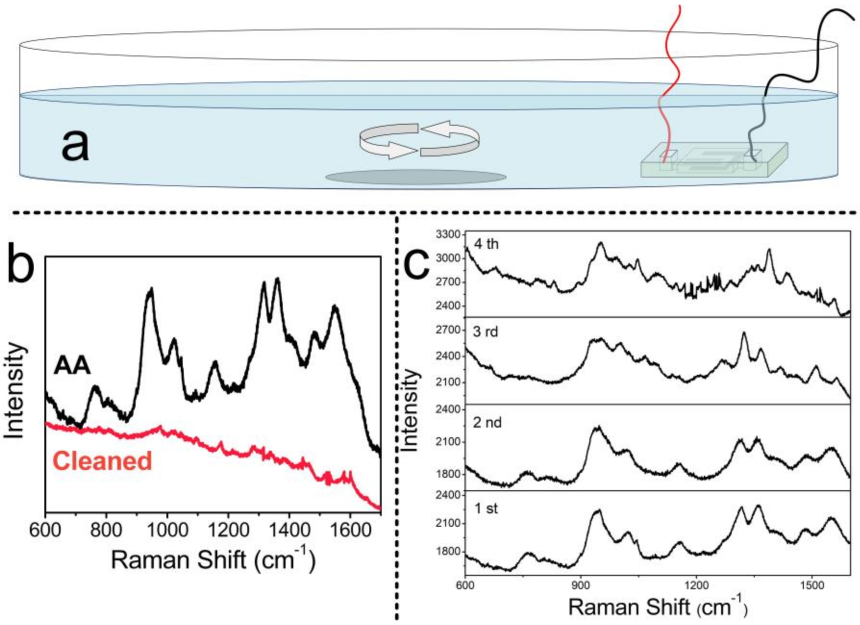

Figure S7. (a) Schematic illustration of cleaning process of microfluidic EC-SERS chip. (b) The SERS spectra of AA using the microfluidic EC-SERS chip and the reused chip after cleaning. (c) The Raman spectra of 1 μM AA by four measurement/cleaning cycles. In the fourth measurements, the Raman signals become unstable.

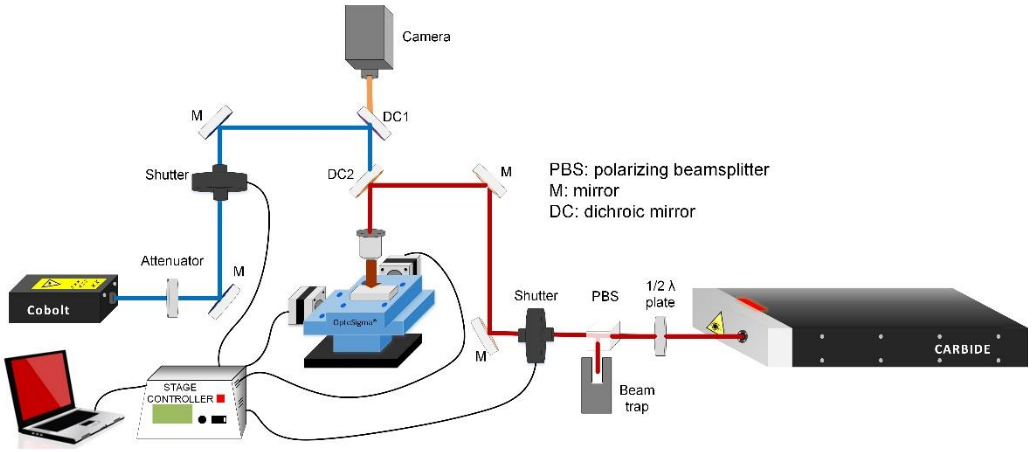

Figure S8. Schematic illustration of hybrid laser system. A 1030 nm fs laser or 405 nm continuous wave laser are focused on the samples using an objective lens.

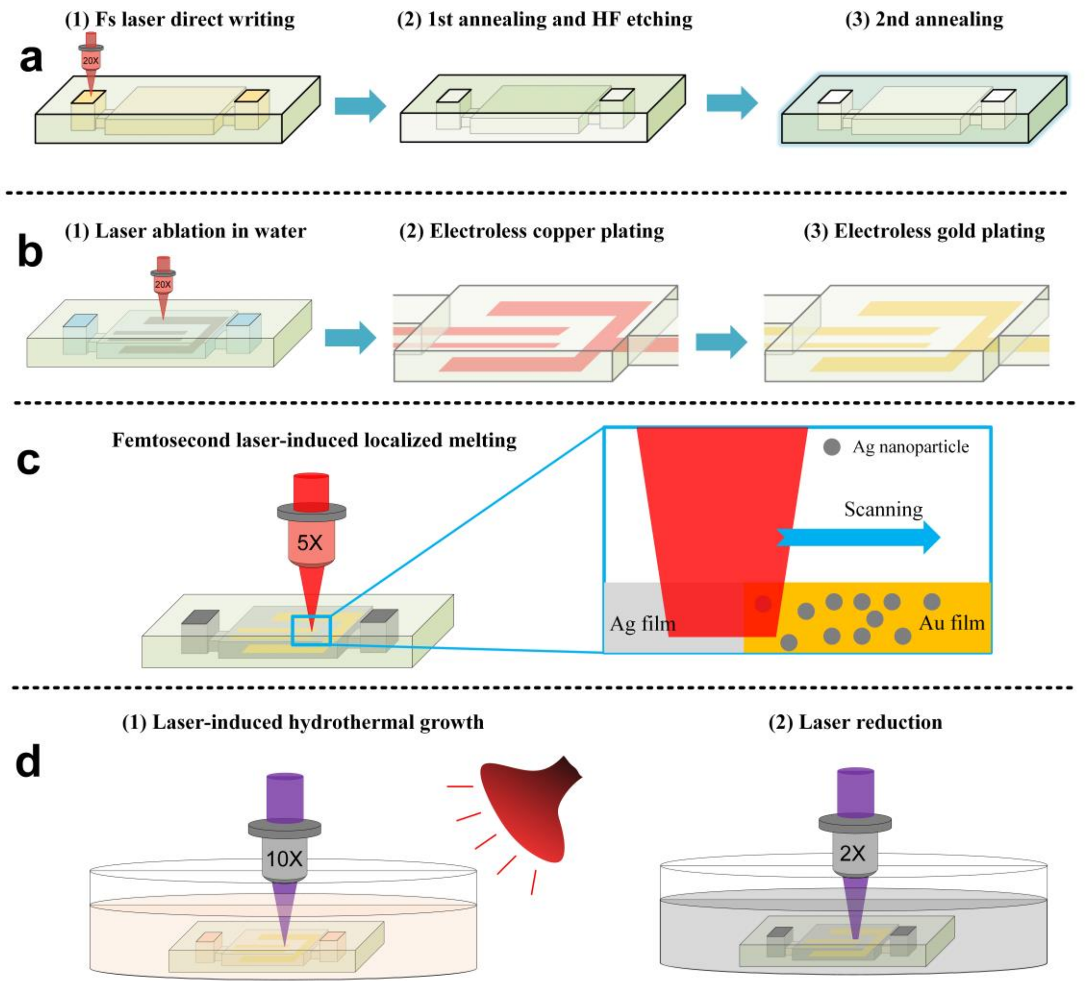

Figure S9. Schematic illustration of the fabrication of microfluidic EC-SERS chip by advanced hybrid laser processing. (a) Glass microfluidic chip fabricated by fs laser-assisted chemical etching. (b) Femtosecond laser-assisted selective metallization of three-electrode system in glass microchamber. (c) Femtosecond laser-induced localized melting of silver nanoparticles on gold film for formation of reference electrode. (d) Formation of working electrode by laser-induced hydrothermal growth of zinc oxide nanowires followed by laser reduction of silver nanoparticles.

Table S1. Comparison of EC-analysis of vitamins in size of electrode, running concentration, and required volume.

**EC-SERS substrate**	**Analyte**	**Size of electrode**	**Concentration**	**Required solution**	**References**
Au modified SPE	B vitamins	12.5 mm^2	10 μM	> 50 μl	[1]
Ag@Au modified ITO electrode	uric acid/ascorbic acid	1 cm^2	50 - 2000 μM	200 μl	[2]
Au modified SPE	folic acid	12.5 mm^2	1 - 50 μM	> 50 μl	[3]
Ag modified Au electrode	flavin		10 mM	> 5 ml	[4]
Ag modified Au electrode	riboflavin		100 μM		[5]
Microfluidic EC-SERS chip	A vitamin	0.01 mm^2	1 μM	< 10 μl	Presented work

References

[1] D. Ibáñez, A. Pérez-Junquera, M. B. González-García, D. Hernández-Santos, P. Fanjul-Bolado, *Talanta* **2020**, *206*, 120190.

[2] G. Bhattacharjee, S. Majumder, D. Senapati, S. Banerjee, B. Satpati, *Mater. Chem. Phys.* **2020**, *239*, 122113.

[3] W. Cheuquepan, S. Hernandez, M. Perez-Estebanez, L. Romay, A. Heras, A. Colina, *J. Electroanal. Chem*. **2021**, *896*, 115288.

[4] M. Abdelsalam, P. N. Bartlett, A. E. Russell, J. J. Baumberg, E. J. Calvo, N. G. Tognalli, A. Fainstein. *Langmuir* **2008**, *24*, 7018.

[5] M. R. Bailey, Z. D. Schultz, *Analyte* **2016**, *141*, 5078.
