# Supplementary figures and images for: Ultraminiaturized Microfluidic Electrochemical Surface‐Enhanced Raman Scattering Chip for Analysis of Neurotransmitters Fabricated by Ship‐in‐a‐Bottle Integration
Source: Small Sci. 2023 Jan 29;3(3):2200093. doi: 10.1002/smsc.202200093 (PMC11935879; doi:10.1002/smsc.202200093)

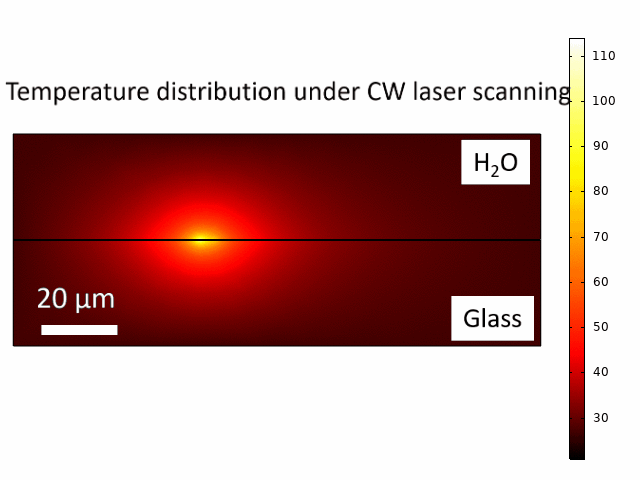

Supplement: Supplementary file 1 — Supplementary Material [file SMSC-3-2200093-s001.zip › smsc202200093-sup-0002-SuppData-S2.gif]
